# A246 EXAMINING BARRIERS TO PHYSICAL ACTIVITY IN PEDIATRIC INFLAMMATORY BOWEL DISEASE PATIENTS

**DOI:** 10.1093/jcag/gwad061.246

**Published:** 2024-02-14

**Authors:** S Morin, M Byra, R Issenman, B Timmons, J Obeid

**Affiliations:** McMaster University, Hamilton, ON, Canada; McMaster University, Hamilton, ON, Canada; McMaster University, Hamilton, ON, Canada; McMaster University, Hamilton, ON, Canada; McMaster University, Hamilton, ON, Canada

## Abstract

**Background:**

Inflammatory bowel disease (IBD) poses significant challenges to pediatric patients, affecting their physical and psychological well-being. Physical activity can be an essential component of managing IBD, yet its adoption among this population remains suboptimal. Understanding the factors that influence physical activity levels in youth with IBD, particularly barriers to engagement, is crucial.

**Aims:**

(1) Describe physical activity levels in pediatric IBD, and (2) assess the relationship between physical activity and barriers to physical activity in youth with IBD.

**Methods:**

We recruited patients between the ages of 7-17 years with a single confirmed diagnosis of IBD. Participants completed a barriers to physical activity questionnaire adapted from Zabinski et al. (2003). Each item was scored on a scale from 1 to 5, to yield a total barriers score and subdomain scores for body-related, convenience, resource, social, fitness, and disease-related barriers; each score presented as an average out of 5. Lower scores indicated lower barrier burden. Participants also wore accelerometers during waking hours for 7 consecutive days to quantify daily average total physical activity (TPA), light physical activity (LPA), and moderate-to-vigorous physical activity (MVPA). Descriptive statistics and multiple regression analyses were used to determine the relationship between physical activity and barriers to physical activity.

**Results:**

Forty-nine youth completed the study (31% females; age: 14.77±1.98 years). Physical activity levels were low (TPA: 146.6±49.5 min/day; LPA: 104.2±33.4 min/day; MVPA: 42.4±21.2 min/day), with only 21% of participants meeting the 24-hour movement guidelines of 60 min/day of MVPA. Total and resource barriers both significantly predicted TPA (total: β=-47.76, p=0.004; resource: β=-48.30, pampersand:003C0.001), LPA (total: β=-29.01, p=0.01; resource: β=-29.68, pampersand:003C0.001), and MVPA (total: β=-18.75, p=0.01; resource: β=-18.62, pampersand:003C0.001). Fitness barriers significantly predicted TPA (β=-41.48, p=0.016) and MVPA (β=-22.01, p=0.008). Body-related, convenience, social, and disease-related barriers did not significantly predict TPA, LPA, or MVPA.

**Conclusions:**

Our findings confirmed that youth with IBD are not engaging in enough physical activity. Importantly, we found those with higher total barrier, resource- and fitness-related barriers presented with 18-48 fewer minutes of daily physical activity. These findings highlight the need for tailored interventions aimed at reducing specific barriers to support physical activity participation in youth with IBD.

**Funding Agencies:**

None

